# Unmet Need for Mental Healthcare in a Population Sample in Sweden: A Cross-Sectional Study of Inequalities Based on Gender, Education, and Country of Birth

**DOI:** 10.1007/s10597-020-00668-7

**Published:** 2020-07-02

**Authors:** Sara Olsson, Gunnel Hensing, Bo Burström, Jesper Löve

**Affiliations:** 1 School of Public Health and Community Medicine, Institute of Medicine, University of Gothenburg, Box 453, 405 30 Gothenburg, Sweden; 2 Department of Global Public Health, Karolinska Institutet, 171 77 Stockholm, Sweden

**Keywords:** Unmet need, Mental health services, Barriers to care, Common mental disorders, Depression, Social inequalities

## Abstract

This cross-sectional study investigated if gender, education, and country of birth were associated with perceived need and unmet need for mental healthcare (i.e., refraining from seeking care, or perceiving care as insufficient when seeking it). Questionnaire and register data from 2008 were collected for 3987 individuals, aged 19–64 years, in a random population-based sample from western Sweden. Descriptive statistics and logistic regression analyses were used. Men were less likely to perceive a need for care than were women, even after adjusting for mental well-being. Men were also less likely to seek care and perceiving care as sufficient. People with secondary education were less likely to seek care than those with university education. There were no statistically significant differences based on country of birth. The observed gender and education-based inequalities increases our understanding of where interventions can be implemented. These inequalities in unmet need for mental healthcare should be targeted by the healthcare system.

## Introduction

Despite the high prevalence of common mental disorders such as depression and anxiety disorders in high-income countries, many of those affected do not seek care (Alonso et al. 2004; Forsell 2006; Johansson et al. 2013; Kessler et al. 2003; Mojtabai 2009; Mojtabai and Olfson 2006; Wallerblad et al. 2012) or do not perceive that they have received sufficient care (Dezetter et al. 2015; Mojtabai 2009). Different healthcare systems provide different opportunities to address this challenge. The Swedish healthcare system is interesting for its extensive universal health coverage that emphasizes equal access to healthcare based on need (Glenngård 2016; Socialdepartementet). This implies that social positions, indicated by, for example, gender, education, and country of birth, should be irrelevant (Westin et al. 2004). However, research from other high-income countries has found that these indicators are associated with perceived need (Codony et al. 2009; Fassaert et al. 2009; Rabinowitz et al. 1999) and unmet need for mental healthcare (Alonso et al. 2004; Codony et al. 2009; ESEMeD/MHEDEA 2000 investigators et al. 2004; Mackenzie et al. 2012; Mojtabai and Olfson 2006). Given the Swedish healthcare system’s aim to reduce the influence of social positions on unmet need for care, more knowledge is needed of how gender, education, and country of birth are associated with perceived need and unmet need for mental healthcare in Sweden.

The definition of need for mental healthcare is debated (Bebbington et al. 1997). The concept is usually divided into clinician-assessed need based on diagnostic criteria versus individuals’ perceived need for care (Rabinowitz et al. 1999). However, studies have identified a discrepancy between “clinical need” and “perceived need” for mental healthcare since not all individuals with a clinical need for care report any such perceived need (Forsell 2006; Meadows et al. 2000). This may be because many of those fulfilling diagnostic criteria for common mental disorders may not require healthcare treatment (Sareen et al. 2013; Vigo et al. 2016) since most of them have mild to moderate conditions (Kessler et al. 2012; OECD 2012). However, treatment may improve symptoms and reduce disease-related disability (Ghio et al. 2015). Even milder conditions may worsen, and they are associated with lower productivity at workplaces, higher absenteeism, and unemployment (Hewlett and Moran 2014).

Most of those with common mental disorders can be effectively treated within primary care (Hewlett and Moran 2014). In Sweden, mental healthcare is provided at low cost in both primary- and specialized-care units (Glenngård 2016). Despite this, a population-based study from Sweden showed that only half of those fulfilling diagnostic criteria for depression and/or anxiety had sought care for related symptoms within the past year (Wallerblad et al. 2012)**.** Studies from Europe and North America found similar patterns: 37–63% of those with probable depressive disorders had sought care in the past year (Alonso et al. 2004; Kessler et al. 2003; Mojtabai 2009; Mojtabai and Olfson 2006). Common reasons for not seeking care are a preference for self-management, low perceived care need, stigma, ignorance, and cost (Fassaert et al. 2009; Mojtabai et al. 2011; Prins et al. 2011). Furthermore, a Swedish study found that among those seeking care for mental health problems, only half received evidence-based treatment (Forsell 2006). Studies from North America found that 35–40% of those seeking care for mental health problems perceived that they received insufficient care (Dezetter et al. 2015; Mojtabai 2009).

In sum, a large proportion of those who could benefit from mental healthcare do not seek care or do not receive sufficient care. However, unmet need for mental healthcare is not randomly distributed in the population, but differs depending on gender, education, and country of birth (Kirmayer et al. 2007; Mackenzie et al. 2012; Mojtabai and Olfson 2006). Gender, education, and country of birth are indicators of an individual´s or group´s social positions within larger social structures that influence access to power and resources (Braveman 2010). Research from high-income countries in Europe and North America has shown that men (Alonso et al. 2004; Mackenzie et al. 2012), those with lower education (Alonso et al. 2004; Codony et al. 2009; Mojtabai and Olfson 2006), and immigrants (Kirmayer et al. 2007) are less likely to seek mental healthcare. There are conflicting findings regarding the association between gender, education, and country of birth and perceived care sufficiency among those seeking mental healthcare (Blenkiron and Hammill 2003; Lang et al. 2005; Lindert et al. 2008; Mojtabai 2009; Whitley et al. 2017). Differences in unmet need for mental healthcare based on social positions are problematic as they may lead to more persistent and severe conditions among some social groups. This is indicated by the higher prevalence of common mental disorders among those with lower education (Fryers et al. 2005), and by the higher suicide rates among men, in particular men with lower education (Crump et al. 2014), and among some groups of immigrants (Hjern and Allebeck 2002; Johansson et al. 1997).

Few studies consider social positions relative to both perceived need for mental healthcare, refraining from seeking care, and perceiving care as insufficient when seeking care. Such studies are important since the pathway to met need for mental healthcare comprises several steps: perceiving a need for care, seeking care, and receiving sufficient care (Forsell 2006; Goldberg and Huxley 2012). Social inequalities may occur in multiple steps on this pathway. In this study, need for mental healthcare was defined as perceiving a need for mental healthcare, and unmet need was defined as refraining from seeking care, or perceiving care as insufficient when seeking it. This study investigated if social positions, using gender, education, and country of birth as indicators, were associated with perceived need and unmet need for mental healthcare in a Swedish population-based sample. The study also investigated whether reasons for not seeking mental healthcare differed based on these indicators. Four research questions were addressed:

1. Was perceiving a need for mental healthcare associated with gender, education, and country of birth?
2. Was not seeking mental healthcare when perceiving a need associated with gender, education, and country of birth?
3. Was perceiving mental healthcare as insufficient when seeking care associated with gender, education, and country of birth?
4. Were there differences according to gender, education, and country of birth in reported reasons for not seeking mental healthcare?

## Methods

### Study Design and Participants

This cross-sectional study was based on Health Assets Project (HAP) data collected in 2008. The target population in HAP comprised the general population in Region Västra Götaland in western Sweden. The region’s 1.7 million urban and rural inhabitants constitute 17% of Sweden’s population. A random general population sample aged 19–64 years was extracted by Statistics Sweden and invited to participate (*n* = 7984). Data were collected using both Statistics Sweden registers and a postal questionnaire in Swedish. The questionnaire asked about self-reported perceived need for mental healthcare, care-seeking, care sufficiency, and reasons for not seeking mental healthcare, as well as about health and sociodemographic factors. Two postal reminders followed up questionnaire distribution resulting in a final general participation rate of 50% (*n* = 4027). Persons born outside Nordic countries had the lowest participation rate (34%), followed by those with the lowest incomes (39%), those in the youngest age group (19–30 years, 41%), men (44%), and those who were unmarried (46%) (Knapstad et al. 2016). Full details of HAP and an extensive non-response analysis are presented elsewhere (Hensing et al. 2011; Knapstad et al. 2016). In this study, 40 of the 4027 respondents were excluded due to missing data for the question about perceived need for mental healthcare: the final study sample comprised 3987 individuals.

### Measures

#### Independent Variables

Gender (male, female) and country of birth (dichotomized into Nordic versus non-Nordic countries based on nine categories: Sweden, other Nordic countries, other European countries, Africa, Asia, North America, South America, Oceania, and others) were based on register data from Statistics Sweden. Education was based on questionnaire data on level of completed education with six response alternatives categorized into primary education or less, secondary education, and university education.

#### Dependent Variables and Operationalization of Unmet Need

All outcome variables were based on questionnaire data. Research questions 1–3 were investigated using the following outcome variables: (1) perceiving a need for mental healthcare, (2) not seeking mental healthcare when perceiving a need, and (3) perceiving mental healthcare as insufficient. Need for mental healthcare was operationalized as perceiving a need for mental healthcare. Unmet need for mental healthcare was operationalized as not seeking mental healthcare when perceiving a need, or perceiving the mental healthcare as insufficient when seeking it. Perceiving a need for mental healthcare and not seeking mental healthcare were measured by asking “Have you at any time felt so mentally unwell that you felt a need to seek care?” with the response alternatives “yes”, “yes, but did not seek” and “no”. Perceiving a need for care was defined as answering “yes” or “yes, but did not seek” versus “no”. Seeking mental healthcare was defined as answering “yes” (sought care) and not seeking care was defined as answering “yes, but did not seek”. Care-seekers were asked where they had sought care with six response alternatives (primary care, psychiatric outpatient care, private physician, private psychologist or psychotherapist, emergency unit, or other). Care-seekers were also asked “Do you think you received the care that you needed?” with the response alternatives “yes” (i.e., sufficient care) and “no” (i.e. insufficient care).

**Fig. 1:**
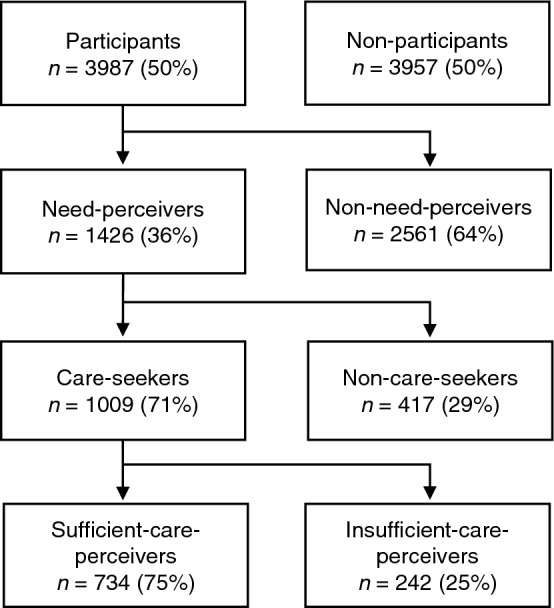
Flow chart of the population-based sample. Categorized by study participation, perceived need for mental healthcare, mental healthcare-seeking, and perceived sufficiency of mental healthcare. Frequency and proportions within category. Dispersed numbers due to *n* = 33 care-seekers with missing data for the question about sufficiency of care

Below, those reporting need are called “need-perceivers” and those not reporting need “non-need-perceivers”. Among need-perceivers, those seeking care are called “care-seekers” and those not seeking care “non-care-seekers”. Among care-seekers, those perceiving that they received insufficient care are called “insufficient-care-perceivers” and those perceiving that they received sufficient care “sufficient-care-perceivers” (Fig. 1).

To investigate the fourth research question, non-care-seekers were asked “Why did you not seek care?” Respondents could choose one or more out of ten response alternatives. Detailed prevalence-per-item data are published elsewhere (Andersson et al. 2014). For this study, reported reasons were merged into six categories: stigma, structural barriers, negative perceptions of healthcare, awaiting recovery, ignorance, and other reasons (Table 4). The categorization emerged through discussion between co-authors combined with review of relevant research (Fassaert et al. 2009; Mojtabai et al. 2011; Prins et al. 2011), and was intended to increase statistical power in the analyses.

#### Covariates

Depression and anxiety disorders have been found to be determinants of perceived need for mental healthcare (Codony et al. 2009), and lower satisfaction with mental healthcare (Wyshak and Barsky 1995). Therefore, mental well-being was used as a control variable to exclude that group differences in previously perceived need for care and perceived care sufficiency were due to differences in current symptoms of depression and anxiety, i.e. clinical need for care. The WHO (Ten) Well-being index (WHO-10) (Bech et al. 1996) comprises ten items covering depression, anxiety, energy, and positive well-being in the previous week. There are four response alternatives for each item (i.e., 0–3, ranging from “never” to “all the time”), giving a total score of 0–30; a lower score indicates lower mental well-being and possibly also clinical need for mental healthcare (Hansson 2009; Hansson et al. 2007). In this study, two cut-offs were used to indicate low mental well-being: ≤ 12, previously found to correspond to depression according to the Major Depression Inventory (Hansson 2009; Hansson et al. 2007); ≤ 8, previously found to correspond to depression according to the Schedules for Clinical Assessment in Neuropsychiatry (Hansson 2009; Hansson et al. 2007). The Swedish version of the WHO-10, used here, has displayed good reliability and validity (Löve et al. 2013).

Persistent illness was measured by questionnaire data on whether the respondent had any persistent disease, illness, or disability followed by a checklist of eleven disease categories (e.g., cardiovascular, neurological, and mental illnesses). Respondents choosing “mental illness” were considered to have persistent mental illness. Those choosing one or more physical illnesses categories were considered to have persistent physical illness. Age, based on register data from Statistics Sweden, was categorized into 19–30, 31–50, and 51–64 years.

### Statistical Analyses

Descriptive statistics were used to outline sociodemographic and health characteristics of need-perceivers, non-care-seekers, and insufficient-care-perceivers. To investigate the first three research questions, proportional differences were assessed using Pearson’s Chi^2^ test (*p* < 0.05). For differences between education groups, proportional differences and 95% confidence intervals (CIs) were calculated (Colton 1974). To investigate the associations between social positions and the three outcomes, logistic regression analyses were conducted by calculating odds ratios (ORs) and 95% CIs. Based on the research questions, the analyses treated somewhat different samples. Research questions 1–3 treated, respectively: (1) the total sample (*N* = 3987), (2) the need-perceivers (*n* = 1426) and (3) the care-seekers (*n* = 976). Bivariate logistic regression analyses were conducted for gender, education, and country of birth relative to the three outcomes. Age was identified as a potential confounder and was controlled for in all multivariable analyses. Multivariable logistic regression analyses were conducted for gender, education, country of birth and age, simultaneously relative to the three outcomes. Mental well-being was identified as a potential moderator for research question one (perceiving a need for care) and three (perceiving the care as insufficient). Therefore, mental well-being was controlled for regarding research questions one and three by conducting sensitivity analyses, by simultaneously adding gender, education, country of birth, age, and mental well-being to the multivariable logistic regression analyses for the two outcomes, respectively. This was conducted for both low mental well-being cut-offs (i.e., ≤ 12 and ≤ 8). Additional sensitivity analyses were conducted where all analyses described above were weighted for differences in participation rates, based on register data on gender, age, country of birth, marital status, income, and rural/urban area, and these results were consistent with those reported below. The results are available upon request. For analyses including variables with missing data, available-case analysis was conducted. Analyses were conducted in IBM SPSS Statistics version 24.

To investigate the fourth research question about differences in reported reasons for not seeking mental healthcare, proportions and their differences (Newcombe and Altman 2000) were calculated relative to gender, education, and country of birth among the non-care-seekers (*n* = 406). For the proportional differences, 95% CIs were calculated using Newcombe’s method (Newcombe and Altman 2000; Vassarstats: Website for Statistical Computation).

## Results

In the total sample (*N* = 3987), 45% were men, 18% had primary education or less, 44% had secondary education, and 9% were born in non-Nordic countries. Of those 3987, 36% had perceived a need for mental healthcare at any time in life (need-perceivers, *n* = 1426): of those, 29% did not seek care (non-care-seekers, *n* = 417); of those seeking care, 25% reported receiving insufficient care (insufficient-care-perceivers, *n* = 242, Fig. 1). In total, 46% of need-perceivers were either non-care-seekers or insufficient-care-perceivers. Of the care-seekers, 45% sought care at primary care, followed by private psychologist or psychotherapist (18%), psychiatric outpatient care (17%), emergency unit (9%), private physician (8%), or other (17%).

Table 1 shows that more need-perceivers than non-need-perceivers were women, university educated (versus secondary educated), born in non-Nordic countries, had current low mental well-being, and persistent mental and physical illness. Among need-perceivers, more non-care-seekers than care-seekers were men, secondary educated (versus primary or university educated), younger, and did not have any persistent mental illness (Table 2). Among care-seekers, more men, people born in non-Nordic countries, young people, those with current low mental well-being, and persistent mental illness, reported receiving insufficient versus sufficient care (Table 2).

**Table 1:** Characteristics of the total sample by perceived need for mental healthcare

	Total sample	Need-perceivers among total sample		
	*N*	*n*	%^a^ (95% CI)	
Total	3987	1426	*36* (34–37)	
Gender				
Men	1775	454	*26* (24–28)	*
Women	2212	972	*44* (42–46)	
Age, years				
19–30	824	298	*36* (33–40)	
31–50	1783	666	*37* (35–40)	
51–64	1380	462	*33* (31–36)	
Education				
Primary or less	722	251	*35* (31–38)	
Secondary	1737	590	*34* (32–36)	*
University	1488	568	*38* (36–41)	
Missing	40			
Country of birth				
Nordic	3609	1273	*35* (34–37)	*
Non-Nordic	378	153	*40* (36–46)	
Mental well-being				
Low, ≤ 12	693	484	*70* (66–73)	*
High, ≥ 13	3058	853	*28* (26–30)	
Missing	236			
Persistent mental illness				
Yes	218	204	*94* (90–96)	*
No	3769	1222	*32* (31–34)	
Persistent physical illness				
Yes	1904	829	*44* (41–46)	*
No	2083	597	*29* (27–31)	

Frequencies (N), proportions (*%*) with 95% confidence intervals (CI), and statistically significant differences between groups (*p* < 0.05)

*Statistically significant difference (*p* < 0.05) based on Pearson’s Chi^2^ test

^a^Proportions, in italics, by row. Valid proportions, missing values excluded

**Table 2:** Characteristics of need-perceivers by not seeking mental healthcare, and care-seekers by perceived insufficiency of mental healthcare

	Need-perceivers	Non-care-seekers among need-perceivers			Care-seekers	Insufficient-care-perceivers among care-seekers		
	*n*	*n*	%^a^ (95% CI)		*n*	*n*	%^a^ (95% CI)	
Total	1426	417	*29* (27–32)		976 ^b^	242	*25* (22–28)	
Gender								
Men	454	163	*36* (32–41)	*	283	83	*29* (24–35)	*
Women	972	254	*26* (23–29)		693	159	*23* (20–26)	
Age, years								
19–30	298	132	*44* (39–50)		162	54	*33* (26–41)	
31–50	666	177	*27* (23–30)	*	471	122	*26* (22–30)	*
51–64	462	108	*23* (20–28)		343	66	*19* (15–28)	
Education								
Primary or less	251	62	*25* (20–31)		176	43	*24* (18–32)	
Secondary	590	212	*36* (32–40)	*	368	89	*24* (20–29)	
University	568	138	*24* (21–28)		420	108	*26* (22–30)	
Country of birth								
Nordic	1273	366	*29* (26–31)		879	210	*24* (21–27)	*
Non-Nordic	153	51	*33* (26–41)		97	32	*33* (24–43)	
Mental well-being								
Low, ≤ 12	484	145	*30* (26–34)		326	113	*35* (30–40)	*
High, ≥ 13	853	248	*29* (26–32)		592	116	*20* (17–23)	
Persistent mental illness								
Yes	204	16	*8* (5–12)	*	182	59	*32* (26–40)	*
No	1222	401	*33* (30–36)		794	183	*23* (20–26)	
Persistent physical illness								
Yes	829	236	*28* (25–32)		573	149	*26* (23–30)	
No	597	181	*30* (27–34)		403	93	*23* (19–28)	

Frequencies (N), proportions (*%*) with 95% confidence intervals (CI), and statistically significant differences between groups (*p* < 0.05)

*Statistically significant difference (*p* < 0.05) based on Pearson’s Chi^2^ test

^a^Proportions, in italics, by row. Valid proportions, missing values excluded

^b^Dispersed numbers due to *n* = 33 care-seekers with missing data for the question about sufficiency of care

### Associations Between Social Positions, Perceived Need, and Unmet Need for Mental Healthcare

#### Perceived Need for Mental Healthcare

As seen in Table 3, men were less likely than women to perceive a need for mental healthcare, even when adjusting for education, country of birth, and age (OR 0.44, 95% CI 0.38–0.50). This result persisted even when adjusting for current mental well-being (using either the ≤ 8 or ≤ 12 cut-off, results available upon request). Moreover, people born outside Nordic countries were more likely to perceive a need for care, when adjusting for gender, education, and age (OR 1.31 95% CI 1.05–1.64). However, this association was not statistically significant when also adjusting for current mental well-being (using either the ≤ 8 or ≤ 12 cut-off, results available upon request). There was no association between education and perceived need for mental healthcare (Table 3).

**Table 3:** Bivariate and multivariable association between social positions and perceived need and unmet need for mental healthcare

Total		Need-perceivers among total sample *n* = 1426/3987		Non-care-seekers among need-perceivers *n* = 417/1426		Insufficient-care-perceivers among care-seekers *n* = 242/976	
		OR	(95% CI)	OR	(95% CI)	OR	(95% CI)
Gender							
Men vs. women	Crude	0.44	(0.38–0.50)	1.58	(1.25–2.01)	1.39	(1.02–1.90)
	Adjusted^a^	0.44	(0.38–0.50)	1.61	(1.26–2.06)	1.40	(1.02–1.92)
Education							
Primary or less vs. university	Crude	0.86	(0.72–1.04)	1.02	(0.72–1.44)	0.93	(0.62–1.40)
	Adjusted^b^	0.97	(0.80–1.18)	1.09	(0.76–1.55)	1.00	(0.65–1.51)
Secondary vs. university	Crude	0.83	(0.72–0.96)	1.75	(1.35–2.26)	0.92	(0.67–1.27)
	Adjusted^b^	0.88	(0.76–1.02)	1.56	(1.20–2.03)	0.82	(0.59–1.15)
Country of birth							
Non-Nordic vs. Nordic	Crude	1.25	(1.01–1.55)	1.24	(0.87–1.77)	1.57	(1.00–2.46)
	Adjusted^c^	1.31	(1.05–1.64)	1.23	(0.85–1.79)	1.53	(0.96–2.42)

Odds ratios (OR) with 95% confidence intervals (CI)

^a^Adjusted for sociodemographic variables: education, country of birth, and age category

^b^Adjusted for sociodemographic variables: gender, country of birth, and age category

^c^Adjusted for sociodemographic variables: gender, education, and age category

#### Not Seeking Mental Healthcare When Perceiving a Need

Men were more likely not to seek mental healthcare than were women, even when adjusting for education, country of birth, and age (OR 1.61, 95% CI 1.26–2.06). People with secondary education were more likely not to seek mental healthcare than were those with university education, even when adjusting for gender, country of birth, and age (OR 1.56, 95% CI 1.20–2.03). There was no association between primary education (versus university education) or country of birth and care-seeking (Table 3).

#### Perceiving that Mental Healthcare was Insufficient

Men were more likely than women to report receiving insufficient care even when adjusting for education, country of birth, and age (OR 1.40, 95% CI 1.02–1.92). When also adjusting for current mental well-being, the association was no longer statistically significant using the higher cut-off, whereas it was statistically significant using the lower cut-off (≤ 12, OR 1.38, 95% CI 0.99–1.92 versus ≤ 8, OR 1.46, 95% CI 1.04–2.03). There was no association between education or country of birth and perceiving care as insufficient (Table 3).

#### Reasons for Not Seeking Mental Healthcare

Of the 417 non-care-seekers, 406 reported one or more reasons for not seeking care (Table 4). The most common category of reasons for not seeking mental healthcare was awaiting recovery (59%), followed by negative perceptions of healthcare (34%), ignorance (29%), stigma (23%), and structural barriers (12%). Negative perceptions of healthcare (e.g., “I did not believe that care would help”) were more common among men than women and among people born outside than within Nordic countries. Ignorance (i.e., “I did not know where to turn for help”) was more common among people with secondary than primary education or less. Structural barriers (e.g., “It was too expensive”) were more often reported by people born outside than within Nordic countries. “It was too far to travel” was not reported by any participant and was excluded from the analysis (Table 4).

**Table 4:** Reasons for not seeking mental healthcare among non-care-seekers by social positions

	Reported any reason	Awaiting recovery^1^	Negative perceptions of healthcare^2^		Ignorance^3^		Stigma^4^	Structural^5^		Other reasons^6^
	*n* = 406^a^	*n* = 240	*n* = 137		*n* = 117		*n* = 95	*n* = 47		*n* = 52
		%^b^ (CI)	%^b^ (CI)		%^b^ (CI)		%^b^ (CI)	%^b^ (CI)		%^b^ (CI)
Total	406	*59* (54–64)	*34* (29–39)		*29* (25–34)		*23* (19–28)	*12* (9–15)		*13* (10–17)
Gender										
Men	158	*58* (50–65)	*40* (32–48)	*	*23* (17–31)		*25* (18–32)	*11* (6–17)		*13* (8–20)
Women	248	*60* (54–66)	*30* (24–36)		*32* (27–39)		*23* (18–28)	*12* (8–17)		*13* (9–17)
Education										
Primary or less	60	*57* (43–69)	*38* (26–52)		*18* (10–30)	*	*17* (8–29)	*13* (6–25)		*12* (5–23)
Secondary	204	*56* (49–63)	*36* (29–43)		*34* (27–41)		*25* (19–31)	*10* (6–15)		*12* (8–18)
University	137	*65* (56–73)	*30* (22–38)		*26* (19–34)		*24* (17–32)	*14* (9–21)		*13* (8–20)
Country of birth										
Nordic	356	*61* (55–66)	*32* (27–37)	*	*29* (24–34)		*23* (19–28)	*10* (7–13)	*	*13* (10–18)
Non-Nordic	50	*48* (34–63)	*46* (32–61)		*28* (16–43)		*24* (13–38)	*24* (13–38)		*8* (2–19)

One or more reasons could be reported. Proportions (*%*) with 95% confidence intervals (CI), and statistically significant differences (*p* < 0.05)

*Statistically significant proportional difference between groups (*p* < 0.05)

^a^Dispersed numbers due to *n* = 11 non-care-seekers with missing data on the question

^b^Proportions, in italics, by row

^1^Awaiting recovery: “It will resolve by itself” (*n* = 240)

^2^Negative perceptions of healthcare: “I did not believe that care would help” (*n* = 133) and/or “I was afraid of being enrolled against my will” (*n* = 8)

^3^Ignorance: “I did not know where to turn for help” (*n* = 117)

^4^Stigma: “I felt ashamed because I felt ill” (*n* = 84) and/or “I was afraid someone would see me when I sought care” (*n* = 28)

^5^Structural: “It was too expensive” (*n* = 44) and/or “The healthcare provider was closed” (*n* = 6) and/or “There was no transportation so I could get to the caregiver” (*n* = 1)

^6^Other reasons: “Other reason” (*n* = 52)

## Discussion

**Fig. 2:**
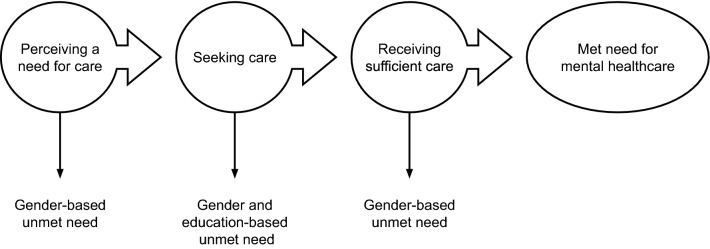
The pathway to met need for mental healthcare with three stages where unmet need may occur. The figure illustrates the study’s main findings of gender- and education-based differences in unmet need for mental healthcare in a population-based sample in Sweden. The findings are based on self-reported data on perceived need for care, care seeking, and perceived sufficiency of care

This study investigated if social positions, as indicated by gender, education, and country of birth, were related to perceived need and unmet need for mental healthcare in Sweden. This study had the advantage of a large population-based sample, also including those who may not fulfil diagnostic criteria of a psychiatric disorder, but may still have an unmet need for mental healthcare. The results showed evident differences in unmet need which is problematic given the Swedish healthcare system’s potential for and ambition to reduce social inequalities in unmet need for care (Glenngård 2016; Socialdepartementet), and the higher risk for more severe conditions among those who do not receive treatment (Ghio et al. 2015; Hewlett and Moran 2014). Gender was the main determinant for both perceived need and unmet need for mental healthcare. Firstly, men were less likely to perceive a need for mental healthcare, than were women, even when they had symptoms corresponding to depression (Hansson 2009; Hansson et al. 2007). Secondarily, men and persons with secondary education were less likely to seek care when perceiving a need than were women and persons with university education. Thirdly, men who sought care were less likely to perceive that they received sufficient care than were women. Based on these results, we observe that the definition of unmet need that was the starting point of the current study, i.e. unmet need only among those who perceive a need for mental healthcare, needs to be revised and also include a lack of perceived need among those with symptoms that indicate a clinical need for mental healthcare. We suggest an extended definition of unmet need, with unmet need at three stages on the pathway to met need for mental healthcare (not perceiving a need for care, not seeking care, and not receiving sufficient care). This definition illustrates the study’s main findings (Fig. 2), and is inspired by previous research by Goldberg and Huxley in 1980 (Goldberg and Huxley 2012), later applied in Swedish research (Forsell 2006). This definition may contribute to both further understanding of and interventions towards social inequalities in unmet need for mental healthcare.

### Gender

That men were less likely to perceive a need for mental healthcare even when they currently had poor mental well-being is in line with previous research from Sweden, Western Europe, and the US (Codony et al. 2009; Forsell 2006; Mojtabai et al. 2002). That men were less likely to seek care is also previously observed (Alonso et al. 2004; Mackenzie et al. 2012). That men more often refrained from seeking care due to negative perceptions of mental healthcare and its effectiveness adds to previous research, although there is some previous support for men having more negative attitudes about the benefit of seeking mental healthcare (Coppens et al. 2013). We also found that men who sought care were more likely to report insufficient care, which adds to previous knowledge. However, this result should be interpreted with caution as the confidence interval was wide and the result contrasts a UK-based study which found no gender difference in satisfaction with mental healthcare (Blenkiron and Hammill 2003).

The above-mentioned differences are interesting given that Sweden is considered as a relatively gender equal country in global comparison (World Economic Forum), with a healthcare system aiming to reduce social inequalities in unmet need for care (Socialdepartementet). These differences are relevant as men’s lower mental healthcare seeking has been suggested to be related to other risk behaviors among men, as high alcohol consumption, and use of violence, as well as men’s higher suicide rates (Rutz and Rihmer 2007). To estimate the magnitude of the problem, longitudinal studies are needed that investigate potential consequences of men’s unmet need for mental healthcare for individuals, and for societies.

Previous research has suggested mental health literacy and masculinity norms as explanations for the influence of gender. For example, studies have found men having more difficulties to identify symptoms of depression (Cotton et al. 2006), indicating poorer mental health literacy. Men may also deny depression to live up to masculinity norms picturing men as strong (Courtenay 2000). These factors may partly explain why we found that men were less likely to perceive a need for care, even when they had symptoms corresponding to depression.

Accordingly, many men with depression may not be in contact with the mental healthcare, and the healthcare system should develop strategies to reach them (Rutz and Rihmer 2007). The healthcare system should also review how its design and ways of communicating may contribute to men’s lower perceived need for care. A potentially lower level of mental health literacy among men might relate to societal factors, e.g. the design and communication of the healthcare system, as health literacy is constructed in interaction with the society (Kindig et al. 2004).

In addition, the barriers observed in the current study may exist or be more emphasised in some groups of men but not in others, due to differences in masculinities (i.e. norms) (Hankivsky 2012). Some groups of men may be more vulnerable for severe consequences, as indicated by the higher suicide rates among men with lower education (Crump et al. 2014). Future research should focus on potential group differences and changes in masculinities and men’s unmet need for mental healthcare, considering that the topic has received increasing attention since our data was collected in 2008 (World Health Organization Europe 2018).

### Education

We observed a social gradient where people with a secondary education were less likely to seek mental healthcare than were people with a university education were. This is in line with previous Swedish (Wallerblad et al. 2012), Canadian, and US observations (Mojtabai and Olfson 2006). These findings possibly reflect the “inverse care law”, i.e., healthcare is most available to those needing it least (Tudor Hart 1971), a worrisome tendency considering the responsibility of the Swedish healthcare system to be of benefit to those needing care the most (Socialdepartementet). The mechanism behind the inverse care law may be multifaceted. For example, research suggests that demand for care is higher among people with higher education and less need (Burström 2009). More educated groups generally have higher levels of health literacy (Paasche-Orlow et al. 2005), potentially driving the demand for care. After our data was collected, a reform criticized for favoring affluent groups with less care need over groups with lower education and greater need, has been implemented in the Swedish primary care system (Burström et al. 2017). Therefore, the observed education-based differences may have increased.

In contrast to the finding above, the expected social gradient in mental healthcare-seeking (Mojtabai and Olfson 2006) was not found in relation to primary education versus university education. However, this might be explained by the higher illness burden among those with a primary education (Muntaner et al. 2013), which could translate into greater care-seeking. Still, greater care-seeking among those with a primary education may not match the higher level of need for care in this group (Burström et al. 2017). Considering the positive effects of receiving treatment for poor mental health (Ghio et al. 2015), we suggest that the Swedish healthcare system should be designed to better benefit those needing care the most, regardless of social positions (Burström 2009). Based on our results, we also suggest that the healthcare system increases acceptability of mental healthcare and improve information about accessing mental healthcare (Forsman and Bomze 2012), especially among those with lower education: We found that 29% of those refraining from seeking mental healthcare reported not knowing where to seek care, this proportion being highest among those with a secondary education (34%).

### Country of Birth

People born outside Nordic countries were more likely to perceive a need for mental healthcare, but the association was no longer statistically significant when controlling for current mental well-being*.* This suggests that the higher likelihood of perceiving a need for mental healthcare is attributable to the poorer mental well-being and general health in this group, previously confirmed within the same study population (Ranjbar et al. 2017). Unlike previous studies (Kirmayer et al. 2007; Lindert et al. 2008), this study found no association between country of birth and not seeking care or perceiving care as insufficient. However, our results should be interpreted cautiously due to some methodological limitations. Firstly, our crude categorization of country of birth as Nordic/non-Nordic may have hidden differences in unmet need. Immigrants are a heterogeneous group: some immigrant groups may face greater barriers to care than others based on their position within Swedish society (Lindert et al. 2008). Secondarily, previous research has found that people of non-Swedish origin have higher likelihoods of both care-seeking and refraining from care-seeking care (Westin et al. 2004). In this study, the questions about mental healthcare-seeking referred to care-seeking at one time point only and could therefore not capture the number of times people refrained from seeking care, i.e. the extent of unmet need for mental healthcare. Based on these limitations, our study may have underestimated differences in unmet need for mental healthcare based on country of birth.

### Methodological Considerations

A strength of this study is the focus on perceived need for mental healthcare in a population-based sample. Many epidemiological studies are based on populations with probable psychiatric disorders, assessed using scales or diagnostic interviews (Dezetter et al. 2015; Forsell 2006; Mackenzie et al. 2012; Mojtabai 2009; Mojtabai and Olfson 2006; Wallerblad et al. 2012), possibly excluding those needing care who do not fulfil diagnostic criteria. Our approach let us highlight discrepancies between perceived need for mental healthcare and clinical need for care in a large populations-based sample, rather than a clinical sample. However, our ability to detect discrepancies was affected by the time periods covered by dependent variables. The questions about perceived mental healthcare need, care-seeking, and perceived sufficiency of care referred to *any time in life*, but we only had data on clinical need for care (i.e., low mental well-being) *at the time of the questionnaire*. However, sensitivity analyses revealed a strong correlation between previously perceived mental healthcare-need and current low mental well-being, a correlation aligned with the often long-standing and recurrent nature of common mental disorders (Kessler et al. 2003). Therefore, we had some ability to investigate whether differences in perceived need for care were due to differences in clinical need for care. Another limitation is that groups facing potentially greater barriers to care, such as men, people with lower income, and people born outside the Nordic countries (Alonso et al. 2004; Kirmayer et al. 2007; Mackenzie et al. 2012; Packness et al. 2017; Westin et al. 2004), had the lowest study participation (Knapstad et al. 2016). This may have led to an underestimation of the influence of these social positions on unmet need for mental healthcare, assuming that participants in these groups were not representative of these groups in the population regarding unmet need. Accordingly, Agerholm et al. found that men and immigrants that did participate in a large population-based study in Sweden had higher healthcare utilization than men and immigrants who did not participate (Agerholm et al. 2016). For this reason, this study may have underestimated the magnitude of social inequalities in unmet need for mental healthcare.

## Conclusion

This study identified gender and education-based inequalities in unmet need for mental healthcare in a population-based sample in Sweden. Gender-based differences in unmet need were found at three stages on the pathway to sufficient mental healthcare: men were less likely to perceive a need for care, seek care, and perceive care as sufficient. Also, persons with secondary education were less likely to seek mental healthcare than those with a university education. This study’s ability to detect social inequalities at multiple steps in the process to met need increases the understanding of where unmet need may occur and where interventions can be implemented. The observed gender and education-based inequalities are worrying given the aim and potential of the Swedish healthcare system to reduce social inequalities by prioritising care to those needing it the most. The healthcare system needs to review how its design and ways of communicating may reinforce social inequalities in unmet need and potentially influence the major barriers found in this study: low perceived need for care, low trust in mental healthcare and its effectiveness, and lack of knowledge of where to seek care.

## References

[CR1] Agerholm J, Bruce D, Burström B (2016). Comparing healthcare utilization among health survey respondents with the total population—Are respondents representative?. BMC Health Services Research.

[CR2] Alonso J, Angermeyer MC, Bernert S, Bruffaerts R, Brugha T, Bryson H (2004). Use of mental health services in Europe: Results from the European Study of the Epidemiology of Mental Disorders (ESEMeD) project. Acta Psychiatrica Scandinavica.

[CR3] Andersson LM, Moore CD, Hensing G, Krantz G, Staland-Nyman C (2014). General self-efficacy and its relationship to self-reported mental illness and barriers to care: A general population study. Community Mental Health Journal.

[CR4] Bebbington P, Marsden L, Brewin C (1997). The need for psychiatric treatment in the general population: The Camberwell Needs for Care Survey. Psychological Medicine.

[CR5] Bech P, Gudex C, Staehr Johansen K (1996). The WHO (Ten) well-being index: Validation in diabetes. Psychotherapy and Psychosomatics.

[CR6] Blenkiron P, Hammill C (2003). What determines patients' satisfaction with their mental health care and quality of life?. Postgraduate Medical Journal.

[CR7] Braveman P (2010). Social conditions, health equity, and human rights. Health and Human Rights.

[CR8] Burström B (2009). Market-oriented, demand-driven health care reforms and equity in health and health care utilization in Sweden. International Journal of Health Services.

[CR9] Burström B, Burström K, Nilsson G, Tomson G, Whitehead M, Winblad U (2017). Equity aspects of the Primary Health Care Choice Reform in Sweden—A scoping review. International Journal for Equity in Health.

[CR10] Codony M, Alonso J, Almansa J, Bernert S, Giovanni de Girolamo M, de Graaf R (2009). Perceived need for mental health care and service use among adults in Western Europe: Results of the ESEMeD project. Psychiatric Services.

[CR11] Colton T (1974). Statistics in medicine.

[CR12] Coppens E, Van Audenhove C, Scheerder G, Arensman E, Coffey C, Costa S (2013). Public attitudes toward depression and help-seeking in four European countries baseline survey prior to the OSPI-Europe intervention. Journal of Affective Disorders.

[CR13] Cotton SM, Wright A, Harris MG, Jorm AF, Mcgorry PD (2006). Influence of gender on mental health literacy in young Australians. Australian & New Zealand Journal of Psychiatry.

[CR14] Courtenay WH (2000). Constructions of masculinity and their influence on men's well-being: A theory of gender and health. Social Science & Medicine.

[CR15] Crump C, Sundquist K, Sundquist J, Winkleby MA (2014). Sociodemographic, psychiatric and somatic risk factors for suicide: A Swedish national cohort study. Psychological Medicine.

[CR16] Dezetter A, Duhoux A, Menear M, Roberge P, Chartrand E, Fournier L (2015). Reasons and determinants for perceiving unmet needs for mental health in primary care in Quebec. Canadian Journal of Psychiatry. Revue Canadienne de Psychiatrie.

[CR17] ESEMeD/MHEDEA 2000 investigators, Alonso, J., Angermeyer, M. C., Bernert, S., Bruffaerts, R., Brugha, T. S. (2004). 12-Month comorbidity patterns and associated factors in Europe: Results from the European Study of the Epidemiology of Mental Disorders (ESEMeD) project. Acta Psychiatrica Scandinavica.

[CR18] Fassaert T, de Wit MA, Tuinebreijer WC, Verhoeff AP, Beekman AT, Dekker J (2009). Perceived need for mental health care among non-western labour migrants. Social Psychiatry and Psychiatric Epidemiology.

[CR19] Forsell Y (2006). The pathway to meeting need for mental health services in Sweden. Psychiatric Services.

[CR20] Forsman L, Bomze S, Backman G (2012). International Human Rights Law and the Right to Health: An overview of legal standards and accountability mechanisms. The right to health: Theory and practice.

[CR21] Fryers T, Melzer D, Jenkins R, Brugha T (2005). The distribution of the common mental disorders: Social inequalities in Europe. Clinical Practice and Epidemiology in Mental Health.

[CR22] Ghio L, Gotelli S, Cervetti A, Respino M, Natta W, Marcenaro M (2015). Duration of untreated depression influences clinical outcomes and disability. Journal of Affective Disorders.

[CR23] Glenngård AH, Mossialos E, Wenzl M, Osborn R, Sarnak D (2016). The Swedish health care system, 2015. 2015 International profiles of health care systems.

[CR24] Goldberg DP, Huxley P (2012). Mental illness in the community: The pathway to psychiatric care.

[CR25] Hankivsky O (2012). Women’s health, men’s health, and gender and health: Implications of intersectionality. Social Science & Medicine.

[CR26] Hansson A (2009). Subjective well-being in an adult Swedish population: Findings from a population-based study.

[CR27] Hansson A, Hillerås P, Forsell Y, Lundberg I (2007). The WHO (Ten) well-being index as a screening instrument for major depression in a population-based sample. European Psychiatry.

[CR28] Hensing G, Holmgren K, Mårdby AC (2011). Harmful alcohol habits were no more common in a sample of newly sick-listed Swedish women and men compared with a random population sample. Alcohol and Alcoholism.

[CR29] Hewlett E, Moran V (2014). Making mental health count: the social and economic costs of neglecting mental health care. OECD Health Policy Studies.

[CR30] Hjern A, Allebeck P (2002). Suicide in first-and second-generation immigrants in Sweden A comparative study. Social Psychiatry and Psychiatric Epidemiology.

[CR31] Johansson LM, Sundquist J, Johansson S-E, Bergman B, Qvist J, Träskman-Bendz L (1997). Suicide among foreign-born minorities and Native Swedes: An epidemiological follow-up study of a defined population. Social Science & Medicine.

[CR32] Johansson R, Carlbring P, Heedman Å, Paxling B, Andersson G (2013). Depression, anxiety and their comorbidity in the Swedish general population: Point prevalence and the effect on health-related quality of life. PeerJ.

[CR33] Kessler RC, Avenevoli S, Costello J, Green JG, Gruber MJ, McLaughlin KA (2012). Severity of 12-month dsm-iv disorders in the national comorbidity survey replication adolescent supplement. Archives of General Psychiatry.

[CR34] Kessler RC, Berglund P, Demler O, Jin R, Koretz D, Merikangas KR (2003). The epidemiology of major depressive disorder: Results from the National Comorbidity Survey Replication (NCS-R). JAMA.

[CR35] Kindig DA, Panzer AM, Nielsen-Bohlman L (2004). Health literacy: A prescription to end confusion.

[CR36] Kirmayer LJ, Weinfeld M, Burgos G, Du Fort GG, Lasry J-C, Young A (2007). Use of health care services for psychological distress by immigrants in an urban multicultural milieu. The Canadian Journal of Psychiatry.

[CR37] Knapstad M, Löve J, Holmgren K, Hensing G, Øverland S (2016). Registry-based analysis of participator representativeness: A source of concern for sickness absence research?. British Medical Journal Open.

[CR38] Lang AJ, Rodgers CS, Moyer R, Laffaye C, Satz LE, Dresselhaus TR, Stein MB (2005). Mental health and satisfaction with primary health care in female patients. Women's Health Issues.

[CR39] Lindert J, Schouler-Ocak M, Heinz A, Priebe S (2008). Mental health, health care utilisation of migrants in Europe. European Psychiatry.

[CR40] Löve J, Andersson L, Moore CD, Hensing G (2013). Psychometric analysis of the Swedish translation of the WHO well-being index. Quality of Life Research.

[CR41] Mackenzie CS, Reynolds K, Cairney J, Streiner DL, Sareen J (2012). Disorder-specific mental health service use for mood and anxiety disorders: Associations with age, sex, and psychiatric comorbidity. Depression and Anxiety.

[CR42] Meadows G, Burgess P, Fossey E, Harvey C (2000). Perceived need for mental health care, findings from the Australian National Survey of Mental Health and Well-being. Psychological Medicine.

[CR43] Mojtabai R (2009). Unmet need for treatment of major depression in the United States. Psychiatric Services.

[CR44] Mojtabai R, Olfson M (2006). Treatment seeking for depression in Canada and the United States. Psychiatric Services.

[CR45] Mojtabai R, Olfson M, Mechanic D (2002). Perceived need and help-seeking in adults with mood, anxiety, or substance use disorders. Archives of General Psychiatry.

[CR46] Mojtabai R, Olfson M, Sampson NA, Jin R, Druss B, Wang PS (2011). Barriers to mental health treatment: Results from the National Comorbidity Survey Replication. Psychological Medicine.

[CR47] Muntaner C, Ng E, Vanroelen C, Christ S, Eaton WW, Aneshensel CS, Phelan JC, Bierman A (2013). Social stratification, social closure, and social class as determinants of mental health disparities. Handbook of the sociology of mental health.

[CR48] Newcombe R, Altman D, Altman D, Machin D, Bryant T, Gardner M (2000). Proportions and their differences. Statistics with Confidence: Confidence intervals and statistical guidelines 2nd edition.

[CR49] OECD. (2012). Sick on the job? Myths and realities about mental health and work. Mental Health and Work. Retrieved April 21, 2020, from 10.1787/9789264124523-en.

[CR50] Paasche-Orlow MK, Parker RM, Gazmararian JA, Nielsen-Bohlman LT, Rudd RR (2005). The prevalence of limited health literacy. Journal of General Internal Medicine.

[CR51] Packness A, Waldorff FB, Christensen RD, Hastrup LH, Simonsen E, Vestergaard M, Halling A (2017). Impact of socioeconomic position and distance on mental health care utilization: A nationwide Danish follow-up study. Social Psychiatry and Psychiatric Epidemiology.

[CR52] Prins M, Meadows G, Bobevski I, Graham A, Verhaak P, van der Meer K (2011). Perceived need for mental health care and barriers to care in the Netherlands and Australia. Social Psychiatry and Psychiatric Epidemiology.

[CR53] Rabinowitz J, Gross R, Feldman D (1999). Correlates of a perceived need for mental health assistance and differences between those who do and do not seek help. Social Psychiatry and Psychiatric Epidemiology.

[CR54] Ranjbar V, Fornazar R, Ascher H, Ekberg-Jansson A, Hensing G (2017). Physical and mental health inequalities between native and immigrant Swedes. International Migration.

[CR55] Rutz W, Rihmer Z (2007). Suicidality in men–practical issues, challenges, solutions. Journal of Men's Health and Gender.

[CR56] Sareen J, Henriksen CA, Stein MB, Afifi TO, Lix LM, Enns MW (2013). Common mental disorder diagnosis and need for treatment are not the same: Findings from a population-based longitudinal survey. Psychological Medicine.

[CR57] Socialdepartementet. *Hälso- och sjukvårdslag [Health and Medical Services Act.]* (2017:30). Retrieved April 21, 2020, from https://www.riksdagen.se/sv/dokument-lagar/dokument/svensk-forfattningssamling/halso--och-sjukvardslag_sfs-2017-30.

[CR58] Tudor Hart J (1971). The inverse care law. The Lancet.

[CR59] Vassarstats: Website for Statistical Computation. The confidence interval for the difference between two independent proportions. Retrieved April 21, 2020, from http://vassarstats.net.

[CR60] Vigo D, Thornicroft G, Atun R (2016). Estimating the true global burden of mental illness. The Lancet Psychiatry.

[CR61] Wallerblad A, Möller J, Forsell Y (2012). Care-seeking pattern among persons with depression and anxiety: A population-based study in Sweden. International Journal of Family Medicine.

[CR62] Westin M, Åhs A, Bränd Persson K, Westerling R (2004). A large proportion of Swedish citizens refrain from seeking medical care—Lack of confidence in the medical services a plausible explanation?. Health Policy.

[CR63] Whitley R, Wang J, Fleury M-J, Liu A, Caron J (2017). Mental health status, health care utilisation, and service satisfaction among immigrants in Montreal: An epidemiological comparison. The Canadian Journal of Psychiatry.

[CR64] World Economic Forum. The Global Gender Gap Report 2018. Retrieved April 21, 2020, from https://www3.weforum.org/docs/WEF_GGGR_2018.pdf.

[CR65] World Health Organization Europe. (2018). *Strategy on the health and well-being of men in the WHO European Region*. Retrieved April 21, 2020, from https://www.euro.who.int/en/publications/abstracts/the-health-and-well-being-of-men-in-the-who-european-region-better-health-through-a-gender-approach-2018.

[CR66] Wyshak G, Barsky A (1995). Satisfaction with and effectiveness of medical care in relation to anxiety and depression: Patient and physician ratings compared. General Hospital Psychiatry.

